# Bottle Aging and Storage of Wines: A Review

**DOI:** 10.3390/molecules26030713

**Published:** 2021-01-29

**Authors:** Javier Echave, Marta Barral, Maria Fraga-Corral, Miguel A. Prieto, Jesus Simal-Gandara

**Affiliations:** 1 Nutrition and Bromatology Group, Analytical and Food Chemistry Department, Faculty of Food Science and Technology, University of Vigo, Ourense Campus, E-32004 Ourense, Spain; ecalja@outlook.es (J.E.); marta_piscis6@hotmail.com (M.B.); mfraga@uvigo.es (M.F.-C.); 2 Centro de Investigação de Montanha (CIMO), Instituto Politécnico de Bragança, Campus de Santa Apolonia, 5300-253 Bragança, Portugal

**Keywords:** wine aging, bottle aging, oxygen permeability, wine storage, wine aroma

## Abstract

Wine is perhaps the most ancient and popular alcoholic beverage worldwide. Winemaking practices involve careful vineyard management alongside controlled alcoholic fermentation and potential aging of the wine in barrels. Afterwards, the wine is placed in bottles and stored or distributed in retail. Yet, it is considered that wine achieves its optimum properties after a certain storage time in the bottle. The main outcome of bottle storage is a decrease of astringency and bitterness, improvement of aroma and a lighter and more stable color. This is due to a series of complex chemical changes of its components revolving around the minimized and controlled passage of oxygen into the bottle. For this matter, antioxidants like sulfur oxide are added to avoid excessive oxidation and consequent degradation of the wine. In the same sense, bottles must be closed with appropriate stoppers and stored in adequate, stable conditions, as the wine may develop unappealing color, aromas and flavors otherwise. In this review, features of bottle aging, relevance of stoppers, involved chemical reactions and storage conditions affecting wine quality will be addressed.

## 1. Introduction

The aging of spirits is a historical practice carried out for millennia, which makes alcoholic beverages and intrinsic element of many human cultures. Of these, wine is one of the most ancient and relevant today in many countries [1]. Wine aging has been improved over the centuries, and with the emergence of new technologies in recent decades, new methods and techniques can be applied to shorten the time of aging, as well as increase wine quality. Wines made from black or pink grapes are generally the ones subjected to the aging process, as they are rich in anthocyanins and other phenolic compounds (PC). This reflects upon the levels of total PC of red wines being among 1–5 g/L and 0.2–0.5 g/L in white wines [2]. Hence, white wines are not commonly subjected to aging, since they are far less resistant to oxidation, excepting some sparkling white wines which are fermented in the barrel and few dry whites [3]. By and large, the most commonly aged are red dry wines [4]. Nevertheless, multiple variations to the involved processes may be found, as there exist a wide variety of tastes for each group of consumers, as well as specific methodologies and selected grapes and/or fermentative yeasts used for some types of wines [5]. The winemaking process fundamentally comprise a careful selection of grape variety, harvesting, grape pressing to obtain wine must, alcoholic fermentation, barrel aging, and bottle storage [6]. The most significant chemical changes will take place during barrel aging and ultimately bottle storage, as along the latter the whole composition of wine is altered.

Generally, barrel-aged wines are stored from 3 to 22 months or even several years. There are different kinds of aging approaches, those using oak (*Quercus* sp.) wood barrels (traditional aging) or those using other vessels made of concrete or steel alongside oak wood pieces (accelerated aging). One or other aging process is selected depending on the grape variety, wine type, and aging desired [7,8]. Nevertheless, these may be subjected to regulations. For example, the use of wood chips for many EU wines labeled with designation of origin is not allowed, whereas the International Organisation of Vine and Wine (OIV) lists specifications to their usage [9,10]. The barrel aging step is also called oxidative aging, as low quantities of oxygen come into contact with the wine. Main chemical reactions that take place are linked to the transfer of oxygen and wood compounds. This oxidation can be performed passively, by oxygen ingress through the gaps of the barrel wood staves and wood micropores, or actively, by the supplementation of oxygen in small quantities during accelerated aging (microoxygenation) [11]. During this step, the wine undergoes controlled oxidation that allows a transfer of volatile (i.e., furfurals, norisoprenoids) and non-volatile compounds (i.e., ellagitannins) from the barrel to the wine and vice versa [12]. Therefore, this stage is crucial since this is the moment in which the unique aromatic outline is developed depending of the type of wood used and time of storage [13]. The most common woods employed for aging wine are obtained from different oak species such as *Quercus alba*, *Q. robur*, or *Q. petrea* but also from other species, known to contain high contents of ellagitannins, such as *Acacia*, *Castanea*, or *Prunus* [7,14,15]. Among the multiple variability of PC found in wood some of the most relevant compounds transferred to the wine during this step are ellagitannins, hydroxybenzoic acids, and hydroxycinnamic acids [12]. Other important reactions taking place during barrel storage are the condensation of tannins and flavonols, aldehyde transference and polymerization of pigments [16]. The degree and extent of these reactions relies upon the time of storage and wood used. Additionally, the practice to “toast” or burn barrel wood or wood pieces used in aging yields further compounds like furans, vanillin (a lignin degradation product) or lactones, albeit it may also result in degradation of ellagitannins and norisoprenoids. This is reliant on the toasting degree (low, medium, high) and the oak species employed [17]. The resulting levels of PC prior to bottling will display an impact on the need bottle aging but also on the overall oxidative stability of bottled wine. Thereafter, wines will respond differently to bottle aging, exhibiting a diverse flavor and aromatic profile. In general terms, barrel aged wines have an astringent and strong flavor, that through bottle storage will evolve towards a more fruity, softer flavor as a result of further oxidation of the wine [18]. Some examples of the few white wines benefitted from aging are typically Chardonnay or Sauvignon Blanc as these grapes are considered not very aromatic; or sparkling white wines like Champagne and Cava, which are fermented on the barrel [6,19]. As such, these types of whites acquire are more complex aroma by barrel aging. Nonetheless, as white wines are accounted for lower levels of PC (i.e., anthocyanins) that are of antioxidant nature, prolonged barrel or bottle storage may pose a quality issue, but a desired process for red or rosé wines [20]. In fact, if the desired white wine is enhanced by the aroma acquired barrel storage, it may acquire ellagitannins from the contact to the wood which also contribute to increase its resistance to oxidation. Another common practice, needed for sparkling white winemaking is aging on lees, whether on the barrel or bottle [21]. Wine shelf-life is a difficult matter to assess as bottles may be stored for long periods of time before consumption, even once the bottle aging phase has been concluded in the winery. As opposed to many foods and beverages, wine generally increases its quality the longer it is preserved in the bottle. This is due to complex chemical reactions that take place in long time term. Most of these reactions are due to the passage of environmental oxygen to the wine, that induce oxidative reactions, triggering further chemical interactions between wine compounds [22]. Typically, aged wine is stored in glass bottles and closed with cork stoppers, through which the oxygen will be transferred. There are other materials that can be used as stoppers with diverse gas transference properties, e.g., thermoplastics or aluminum. These materials will determine the oxygen transfer rate (OTR). However, even though the selection of the stopper is going to provide different oxidation degrees to wine, it is not the only factor responsible for the wine aging in the bottle. The most important condition of the susceptibility of the wine towards oxidation in the bottle heavily relies on their phenolic composition. The PC content of wine depends on the grape origin, characteristics of barrel aging and aging conditions. Besides, environmental parameters of storage such as type of closure, temperature, humidity, or exposure to light are going to strongly impact in the development of the wine aging bottle [23]. This diverse resilience of wines against oxidation also determines the choice of the closure. Aged red wines will require a higher OTR in order to ensure bottle oxidation, thus the stoppers are frequently made of natural cork or cork composites. Synthetic stoppers may also be used but as they tend to have higher permeability to oxygen (excepting screw caps), they are less frequently used in bottle aging as its use may impair accelerated/premature oxidation of the wine. The reactions induced by oxygen ingress includes polymerization of pigments, condensation of tannins, formation of new aromatic compounds, and degradation of molecules that lead to undesired aromas and off-flavors. These reactions take place over time, meaning the wine does not stay chemically still through the storage. Some perceivable changes of wine by storage in the bottle include darkening of color, increased fruit flavor, lower astringent and “reductive” flavor, or softer mouthfeel [18]. Hence the bottle acts as an active aging vessel. Altogether, these chemical changes will have a positive impact on the wine qualities. However, non-optimum storage conditions, faults on the qualities of the stopper or an excessive storage time can result in the development of undesirable chemicals and in some cases, even make the wine unfit for consumption [24]. The key aspects, relevance and outcomes of bottle storage of wines will be addressed in the following sections.

## 2. Influence of Closure

**Figure 1:**
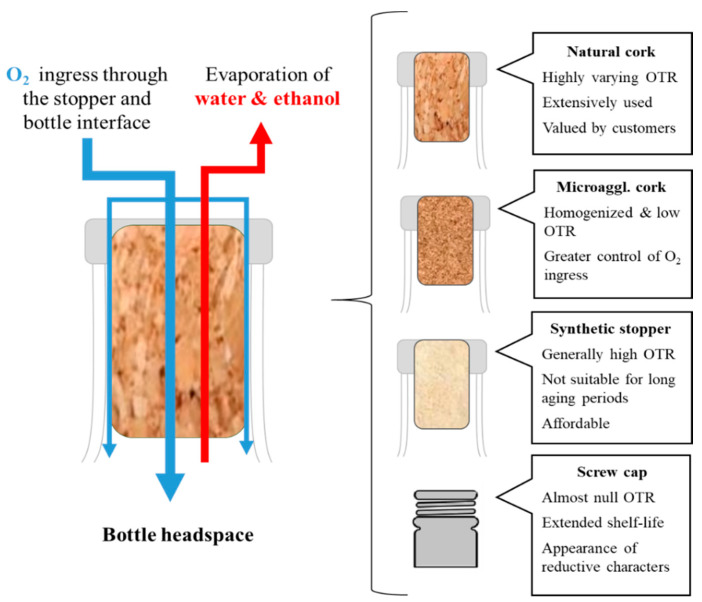
Ingress of oxygen to the bottle through the stopper and characteristics of stoppers.

Once the oxidative aging process has concluded, wine is placed in glass bottles of varying volume (generally containing 0.75 L of wine). The vessel/wine volume ratio is relevant, as it contributes to shape the resistance to oxidation, as well as the available gaseous phase in the bottle headspace [25]. For this matter, under the same storage conditions and time, an extended oxidation is observed in bottles of 0.375 L than in 0.75 L [24]. Although wine can be stored in plastic bottles or plastic/cardboard containers (Bag in Box), glass remains to be the main packaging material used [26,27]. As glass is a hermetic material, the passage of oxygen is only possible through the stopper. In this sense, the stopper of choice can make a difference on the transference of oxygen to the bottled wine, as the porosity of the material used directly affects this parameter [28]. As the stopper is generally gas-porous, it acts as a permeable barrier for different gases, such as alcohol or water vapors from the wine that may be dissipated out of the bottle (Figure 1). Regarding oxygen, it comes into contact with the wine along several steps in winemaking and bottling process, hence when wine is bottled it already contains dissolved oxygen. Besides, after wine has been bottled oxygen will be present in the headspace [29]. To better control the storage and aging, oxygen in the headspace can be evaporated by vacuum and replaced by an inert gas, such as nitrogen. This procedure also avoids pressure difficulties when the bottle is opened and minimize evaporation of water and alcohol [30]. The occupation of the headspace with another gas saturates gas pressure in the bottle and hinders the aging process since it results in negative tones for some wines. Nevertheless, when bottle aging is required to enhance flavor and aroma of wine, oxygen can ingress into the bottle through the stopper.

### Types of Closure

As mentioned, a great number of studies have determined that stopper characteristics greatly influence the bottle aging process and preservation of the wine [28,31,32]. Oxygen may access the bottle by two different mechanisms: By permeation and diffusion. Permeation depends on the gas pressure in the bottle, but this entry pathway can be partially countered by introducing an inert gas, like nitrogen or in the case of sparkling wine, the contained carbon dioxide. This process is less feasible to happen in isothermal conditions, that is why is so important to store wine bottles at steady temperature [33]. The diffusion pathway relies on the oxygen concentration gradient and can occur against pressure gradient. Thus, despite of measures taken to lower oxygen transference to the wine, oxygen ingress may occur anyway albeit at lowered rates that allow an extended control over the aging process [34]. Both these routes hinge on the transmission properties of the closure, hence the stopper plays a critical role on modulating oxidation of the stored wine, based on its oxygen permeability. Moreover, gaseous exchange may occur not only through the stopper, but also via the stopper–glass interface, which needs to be tightly closed [35]. In fact, this path of entry for oxygen has been found to be a major issue when stoppers loose tightness over time or inadequate storage conditions [28]. For this reason, over the years, winemakers have analyzed the performance of different closures and the physical alterations that improve their enclosing properties. The usual closure system consists of cork stoppers. However, cork may be subjected to different treatments of its structural conformation and particle size that leads it to have different permeability to oxygen. Wineries use other materials to enclose their bottles as well, like synthetic composites, screw caps made of aluminum with a thermoplastic layer, or even caps made solely of polyethylene [28]. Yet, porous stoppers remain the most used, since they allow a proper aging of the wine, while screw caps are almost airtight and greatly limit the ingress of oxygen. In turn, the very low oxygen ingress allowed by screw caps affects the wine chemical environment and yield more reductive characters [36]. In contrast, polyethylene caps are excessively porous, yielding a premature oxidation of the wine [37]. When made of permeable materials (cork, synthetics), stoppers require a mechanical compression that will additionally reduce their permeability in their contact interface with the glass, yet not the permeability of the stopper [35]. The size of the stopper is relevant too, as it determines its available surface (diameter) and filter thickness (length) [38]. A stopper size may vary from 22–24 mm of diameter to 28–46 mm of length, cork stoppers usually being the larger [35,39]. Cork is composed of suberin, lignin, cellulose, and hemicellulose along with minor quantities of tannins or waxes. Synthetic stoppers are commonly made of low density polyethylene if they are produced by molding process, either styrene–butadiene–styrene or styrene–ethylene–butylene–styrene in a molding process or rather a mixture of low density polyethylene and ethylene vinyl acetate [38]. The mechanical and chemical properties of these materials make them convenient for their use as microporous closures.

Regarding their structure, stoppers show diverse particle size that will later influence their permeability to oxygen. Cork stoppers may be extracted from cork oak bark as a single piece (natural cork), macroagglomerated particles (2–8 mm size) or microagglomerated particles (≤2 mm) jointed together as cork composites by blended with polyurethanes and isocyanates [38]. Microagglomerated stoppers are also called technical stoppers. Besides this, in the case of sparkling wines, the stopper is usually a multilayered cylinder with a central body of natural cork or macroagglomerated cork and two microagglomerated disks at each end. This configuration allows for an improved control over the gas transference from and to the wine [40]. There are several methods and measures to address the transference of gases such as diffusion coefficient or permeability. However, the most used and practical value is OTR [41] that may be calculated by physical measures of the stopper properties, i.e., inferring from their effective diffusion or rather indirectly by determining the oxygen concentration in the bottle or the degree of degradation of compounds in the wine or even apparent characteristics (i.e., yellow color by measuring absorbance at 420 nm or chemiluminescence) [34,42]. The units of OTR are usually given as mg or ml of O_2_ per day, month or year [29]. This allows not only to determine the passage of oxygen, but also to easily conceive what amount may be added to the wine through bottle aging and best fits each type of wine. In turn, a more efficient selection of the stopper and closure used is possible [33]. As cork is a natural material, it is also heterogeneous and natural cork stoppers show a broad spectrum of OTR, since the microscopic structure of its cells varies greatly. Agglomerated cork, or technical stoppers have tightened range of OTR, as they are more homogeneous [43]. Yet, the OTR values may still differ, for the material permeability is still linked to the microscopic structure despite a homogenization of particle size [38]. In summary, evidence suggests that in general terms, natural cork stoppers have a varying yet good OTR that can be homogenized by microagglomeration while synthetic stoppers offer in many cases excessive OTR for long-aging wines. On the other hand, while screw caps may be a good option to preserve wine in non-optimal conditions of storage, are prone to induce the development of “reductive” characters [44]. A general overview on the OTR values of stoppers is presented in Table 1.

Stoppers made of cork are commonly subjected to physico-chemical treatments to improve their properties and sanitize them, preventing the transference of undesired compounds to the bottled wine. The foremost method, used at industrial scale, is CO_2_ supercritical treatment. It has proven to be very successful to preserve the wine without negatively affecting permeability of cork stoppers [51]. On the other hand, stopper surface treatments are done for various purposes, like ease the extraction of the stopper or avoid liquid leakage. On top of that, surface coatings have also been found to lower the oxygen diffusion through the stopper–glass interface of the bottleneck [43]. Such surface treatments are carried on cork stoppers and are commonly made with paraffin waxes or silicon [52]. Another way to lower gas permeability is to cover the closure with a metallic or plastic layer (capsule) over the stopper, while this operation is also done to protect the stopper during handling and transportation [6,53]. Nevertheless, encapsulation of the closure has proven to be an effective measure to limit excessive oxidation and preserve wines from undesired aromas (i.e., haloanisoles), extending their shelf-life [54].

The contact with the wine and environmental moisture can affect the permeability of cork to oxygen, which is a common feature of filters. Humidity retained in the cork pores affects its mechanical properties, which in turn, alter the permeability. Yet, the absorption capabilities of the stopper are heavily reliant on temperature [55]. Synthetic stoppers, like those made from expanded polyethylene, generally show a higher permeability in comparison with cork. This is a widely known fact, extensively reported in scientific literature but more pronounced in long periods of storage [38]. For example, a study carried out by Silva et al. measuring oxidation of wines after 2 years of storage found that wines enclosed with synthetic stoppers showed greatly higher levels of oxidative markers in comparison to stoppers made of cork [56]. Extruded synthetic stoppers are reported to be more permeable to oxygen in comparison to natural or technical cork, showing more oxidative characters when compared to cork stoppers in the same aging time [31]. Moreover, synthetic stoppers tend to harden over time, loosing tight in the stopper–glass interface, which may result in a premature oxidation [57]. Still, synthetic stoppers could be valuable for young wines or those simply needing short aging periods. Conversely, albeit screw caps frequently contribute to the development of “reductive” aromas, also heavily minimize oxidative degradation of the wine [47]. This can be of interest for wines more sensible to oxidation and expected to be consumed in a short period after bottling, as is the general case of white wines.

Although synthetic materials or alternatives to cork offer some benefits like their affordability and absence of off-flavor compounds; natural cork closures remain the most popular for their presence is considered a quality feature among consumers, whereas synthetic stoppers are generally associated with “cheaper” or “lesser” wines [58]. The higher permeability to oxygen that synthetic stoppers display over time also tends to make them less preferred. Yet, it should be considered that many researchers in the field consider that role and influence of many parameters and materials not fully determined, as in the case of stoppers [28,38]. That explains why many successful wineries still face unpredicted issues in their products such as faults and taints that would be more easily controlled in other foods and beverages [59]. Nevertheless, there is an ever-growing interest on defining key winemaking parameters in order to refine and hold more control over the final product quality. For this matter, research in this field has sprouted in recent years.

## 3. Oxidative Stability

Oxidation in wines may occur as enzymatic and non-enzymatic oxidation. Enzymatic oxidation happens almost entirely in wine must during pressing and alcoholic fermentation by a wide variety of oxidoreductases (i.e., laccase, catechol-oxidase, and monophenol monoxigenase) [2]. Hence, the oxidation mechanism that derives in wine changes during bottle storage will be non-enzymatic, involving the degradation of PC by oxygen. It is widely accepted that the oxidative chain-reaction must be catalyzed by metallic ions, namely and Fe, Cu, and Mn, of which iron is the main actor [60,61]. The levels of these metals in wine rely upon the grape variety, growing conditions and vinification techniques (grape pressing, must mix, filtering, among others) [62]. As demonstrated by Danilewicz and Wallbridge, chemical removal of iron with potassium ferrocyanide largely reduced peroxidation and degradation of antioxidants, pointing iron as the major inducer of oxidation [63]. Moreover, the interactions between iron and copper indicate that copper further catalyzes the oxidation process and oxidative balance of iron, but it is a weak catalyst of oxidation itself [64]. After transference, oxygen is present in the headspace of the bottle and diluted in the wine. As it reacts with iron and PC, oxygen takes new forms as reactive oxygen species, of which the most preeminent in wine because of its acid (≈3.5) pH, is hydrogen peroxide (H_2_O_2_), hydroperoxyl radical (HO_2_^−^) and hydroxyl radical (HO^−^) [60].

It has been stated that oxidative reactions will alter in different ways each wine, and this is because of their somewhat diverse PC composition, which directly influences the oxidative balance of the wine and in turn, arbitrates the need and convenience of bottle aging [65]. PC in wine tend to be classified as flavonoid PC and non-flavonoid PC. Flavonoid PC intuitively refer to flavonoid-derivated PC, which can be divided in flavonols and flavan-3-ols. In wine, the main flavonols are quercetin, myricetin and kaempferol, while the main flavan-3-ol derivatives are (+)-catechin and (+)-epicatechin [2]. Non-flavonoid PC englobes a vast range of phenolic acids, namely derivates of benzoic and cinnamic acid, phenolic alcohols, or stilbenes, among other minor groups [66]. Some relevant non-flavonoid PC of interest are hydroxycinnamic acid, gallic acid, vanillic acid, caftaric acid, p-coumaric acid, guaicol, or vinylphenol [2]. Anthocyanins and anthocyanin-derived compounds are classified as pigments, being responsible for the red color of wine. Since anthocyanins are polyphenols, they will act as antioxidants in a similar manner as other PC. Wine tannins may include condensed tannins (built upon polymerized flavan-3-ol subunits) that come from the grape, but a barrel-aged wine will also show hydrolysable tannins; constituted by galloyl or ellagic acid moieties that are transferred from the barrel’s wood [51].

**Figure 2:**
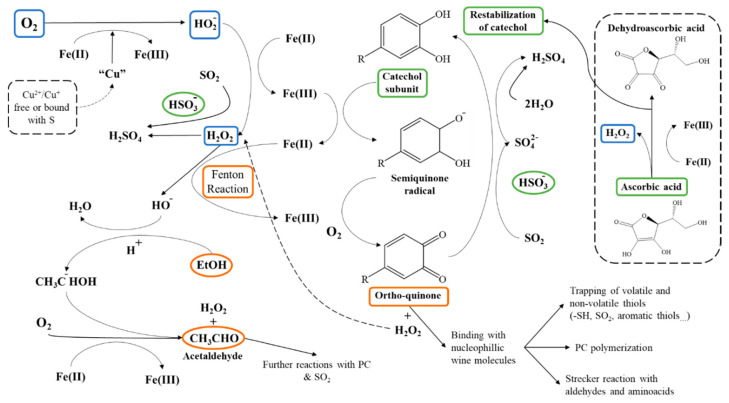
Overview of chemical interactions between SO_2_, metallic ions, quinones and induced subsequent reactions. Ethanol is the main alcohol of wine that reacts to acetaldehyde. Peroxide resulting from the oxidation of ortho-quinones further contributes to formation of acetaldehyde. Ascorbic acid will act as antioxidant and re-stabilizer of catechol subunits until depletion. Adapted [72,73,74].

The transition reaction between ferrous (Fe^2+^) and ferric (Fe^3+^) ions leads to the formation of ${\mathrm{HO}}_{2}^{-}$, then to H_2_O_2_ and HO^−^ in the Fenton Reaction [67]. Ethanol, the major alcohol in wine, is then oxidized to acetaldehyde, which contributes to the “oxidized” aroma of the wine and is used as a marker of oxidative status of bottled wine [64]. Transition between Fe^3+^ and Fe^2+^ also oxidizes PC, producing semi-quinones that oxidize to ortho-quinones by accepting oxygen [60]. Quinones are a wide group of chemicals that share a benzoic ring with two ester groups in ortho- position and a radical of varying structure. The ortho-ester phenolic ring forms from the oxidation of the catechol and galloyl subunits of PC. These subunits are preeminently present in the main PC constituents of wine: Flavonoids, flavan-3-ols, and anthocyanins, alongside gallic and caffeic acids [68,69]. Quinones are highly electrophilic molecules and bind with nucleophilic compounds like volatile and non-volatile thiols, oxidize other PC, induce Strecker degradation of amino acids and “de novo” production of undesired aldehydes [70]. The results of these interactions include the loss of aroma (volatile thiols), color (anthocyanins), and surge of undesired flavors (aldehydes) [18,68]. Additionally, oxidation of semi-quinones to ortho-quinones also results in the formation of H_2_O_2_, providing more available substrate to be oxidized to HO^−^ and then producing more acetaldehyde in the Fenton Reaction. Therefore, ortho-quinones are one of the main agents implied in maintaining the oxidative reaction once initiated by iron [71]. The complex interactions among oxidative and antioxidant chemicals are summarized in Figure 2.

In order to ensure oxidation stability and avoid microbial spoilage of the wine, sulfur dioxide (SO_2_) is routinely added throughout winemaking and prior to bottling [59]. Free SO_2_ exists primarily as bisulfite anion (HSO_3_^−^) at wine pH and is the first chemical to show degradation by oxygen to sulfate (SO_4_^2−^) and then sulfuric acid (H_2_SO_4_) [6]. This explains that its concentration levels have been traditionally used as markers of the oxidation progress of the wine, i.e., by chemical titration, as recognized by the International Organization of Vine and Wine (OIV) [75]. However, SO_2_ does not directly interact with oxygen, but with the resulting H_2_O_2_ and quinones [60]. In this sense, SO_2_, in the form of HSO_3_^−^ helps revert PC to a stable form and contributes to modulate the available reactive quinones [76]. Yet, SO_2_ comes with some drawbacks as its toxicity, potential off-flavors, maximum legal limits based on its possible allergen properties and that its degradation leads to the formation of sulfuric acid, increasing the total acidity of the wine [77]. Another possible way to increase antioxidants in the wine is aging the wine on lees (a mixture of yeasts, lactic acid bacteria and precipitates like tartrate) left after alcoholic fermentation and prior to bottling. This may provide the wine with additional substances regarding its aromatic profile, but also with glutathione, which is a sulfated antioxidant produced by yeasts during fermentation [78]. In fact, adding pure glutathione has been proposed as an alternative exogenous antioxidant for replacing SO_2_, showing similar results on antioxidant activity [18]. Aging on lees is in fact an extended technique in winemaking and required for the production of sparkling wines since, as mentioned, these provide this antioxidant, as well as mannoproteins and additional aromatic molecules yielded from the autolysis of yeasts conforming the lees [79]. Moreover, aging on lees has proven to reduce the presence of undesirable aromatic compounds like 4-ethylphenol and procyanidins; thus improving general aromatic profile and oxidative stability, but also erasing desirable aromatics as 4-ethylguaicol, as a result of these components adhering to the yeasts cell walls [78]. This practice is being increasingly employed, but is also a traditional method of aging wines, like Sherries or Ports as it contributes to the stabilization of pigments and provide them with their characteristic aroma and flavor [80]. Yet, lees may also produce biogenic amines, hazardous chemicals that can make the wine unfit for consumption. In order to avoid the possible undesirable side effects of lees, other alternatives to obtain similar effects may be adding yeasts hydrolysates, pure glutathione as well as modulating the time of aging on lees [7]. Nonetheless, this method is being progressively more used to ensure wine oxidative stability. Other significant compounds implicated in the oxidative stability are ascorbic acid and tartaric acid.

Ascorbic acid is a well-established antioxidant capable of greatly improving the oxidative resilience of the wine. It is used as added as an antioxidant when winemakers desire to use as little SO_2_ as possible. Experimental data shows that its antioxidant activity is dose-dependent, since at low concentrations it can act as a pro-oxidant molecule, and addition of SO_2_ is still required [81]. Yet, added at higher concentrations (≈ 45–90 mg/L) acts as a powerful antioxidant [82]. If added to the wine, ascorbic acid will be preferentially degraded to dehydroascorbic acid to reduce quinones. Once depleted, SO_2_ will be the main antioxidant able to reverse ortho-quinones to the catechol form [83]. Although the antioxidant effect by the addition of ascorbic acid is not apparent in short-term storage, it can sensibly lower the surge of oxidized aromas in longer storages [83]. Dehydroascorbic acid degrades into xylosone, further degraded to 2-furoic acid and 3-hydroxy-2-pyrone [71]. Xylosone is especially relevant, since also acts as precursor intermediate with (+)-catechin in the formation of xanthylium cations that stabilize to xanthylium salts [84]. Xanthylium salts, as will be addressed in following sections, are anthocyanin and catechin-derived pigments formed through various mechanisms that exhibit a yellow color [85]. This color change may worsen color appearance of red wines but also greatly impact appearance of white wines [81]. Tartaric acid on the other hand, is present in the grape and is also carried on to the wine. The most abundant acid in wines alongside malic and citric acid, it is responsible for the acidic flavor of wines, being in higher concentrations in white wines than in red ones [86]. Hence, its levels are measured as titratable acidity to determine the acid flavor of the wine [44]. Besides, it is known that tartaric acid is able to quelate the ferric ion, capturing it and thereafter lowering iron ions available to induce oxidation [87]. Although some of this tartrate conjugate precipitates during barrel aging, most of its concentration is carried to the bottled wine. This is what causes the formation of visible tartrate precipitates in the bottle, considered unappealing in white wines [68]. But most importantly, tartaric acid may also be oxidized yielding glyoxylic acid, which then bonds with the A ring of (+)-catechin resulting in xanthylium cations after subsequent reactions. Hence, tartaric acid is potentially involved in undesired color changes in wines [88].

Altogether, oxidative stability of wines relies on the composition and concentration of its antioxidants, while their behavior and negative effects of their degradation products will ultimately depend on the OTR during storage. Nonetheless, several specific pathways and equilibrium mechanisms are yet to be identified.

## 4. Desired Chemical Changes

Desired changes comprise transformation of wine PC, namely hydroxybenzoic acids, hydroxycinnamic acids, flavonoids, anthocyanins, and tannins. Additionally, the formation of certain aromatic (volatile) thiols and aldehydes strongly contribute to the fine aroma and flavor of aged wines. Major relevant compounds formed because of bottle aging are summarized in Table 2.

### 4.1. Anthocyanins

**Figure 3:**
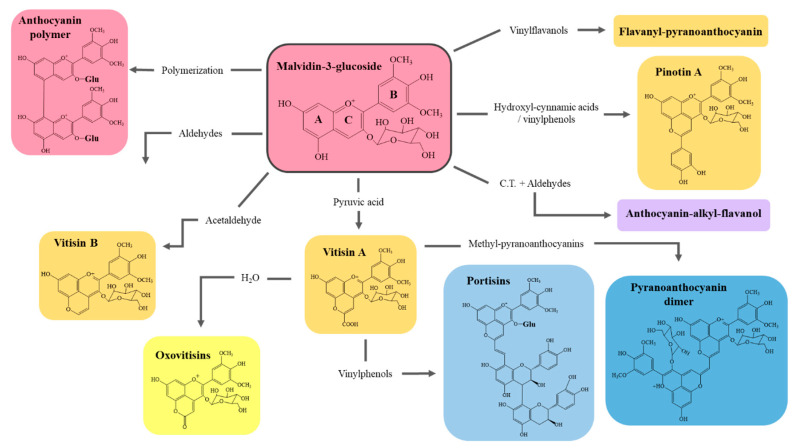
Overview of the general structure of anthocyanins and formation of pyroanthocyanins, representing their coloration in wine acidic medium. C.T.: Condensed tannins. Adapted [113,114].

Anthocyanins are the major grape pigments in red and black grapes and the compounds responsible for color in red wine. They are formed in pigmented grape skin from catechin and epicatechin Most importantly, aging and storage transforms the wine color from dark to bright red by alterations and polymerizations of these compounds [111]. Anthocyanins can be present in different forms depending on medium pH, as bright red flavylium cation form, colorless carbinol form or purple-like quinoidal form. These forms appear in shifting concentrations and chemical equilibrium among them [89]. At wine acidic pH, the main form would be colorless carbinol, but given the reactivity of wine components and the slow oxidative process it is subjected, these anthocyanins evolve towards more stable structures. Among the diversity of anthocyanins present, the most relevant are delphinidin, cyanidin, petunidin, peonidin and malvidin, which are derivatives of 3-*O*-glucosidic anthocyanins [4]. Of these, malvidin-based are predominant and the ones that give place to new, more stable pigments formed in aging, being malvidin 3-glucoside the most representative building block of new pigments [112]. Newly formed anthocyanin pigments may be the result of several interactions with other wine molecules like aldehydes, acetaldehyde, or flavanols (condensed tannins) [96]. Yet, smaller molecules like phenolic acids (mainly hydroxycinnamic acids) and aromatic PC obtained from barrel storage like guaiacol or syringol act as precursor in the chemical changes that anthocyanins are subjected to [113]. These pigments of varying structure show enhanced colors and highly improved stability towards chemical and pH changes in the wine, making wine color much more stable to possible alterations [114]. A notable effect of this is that wine color will be resistant to bleaching caused by SO_2_, a main property of this antioxidant [115]. Pigments derived from anthocyanins usually result in pyranoanthocyanins. Their name comes from the pyranic ring (D) formed between C_4_-C_5_ of the anthocyanidin base unit and from which can stem different radicals, while also being a polymerization binding site (Figure 3) [114].

The first pyranoanthocyanins identified in red wine were vitisin A and vitisin B, which are formed by a condensation reaction with pyruvic acid or cycloaddition with acetaldehyde, respectively [115]. Vitisins are the main anthocyanin-derived pigments in wine, with a bright red-orange color and cause the “clarification” of wine color [116]. Derived from vitisin A, yellow pyranoanthocyanins called oxovitisins have been described, in which the aldehyde radical in the D ring is substituted by an ester through hydrolysis reaction [98]. Another relevant group derived from vitisin A are portisins, which receive their name from being first detected in Port wines [112]. These pigments provide a dark-bluish color [92]. The structure of these compounds is composed by a pyranomalvidin linked to a flavanol through a vinyl group, in which the radical of the pyranomalvidin in the C_3_ may be a glucose (portisins A,C), as in vitisin A, or a coumarouylglucose (portisin B) [93]. Conversely, other relevant group of pyranoanthocyanins are pinotins, first found in Pinotage wines [91]. Pinotin A is the representative pigment of this group and derivates from it are labeled as pinotin A like pigments [24]. Besides these mentioned groups, many combinations of anthocyanins with other wine molecules, foremost PC, are present and relevant in their contribution to the wine’s color. This the case of flavan-3-ol-anthocyanin adducts, alkyl-anthocyanin adducts or flavanyl-pyranoanthocyanins, among others [89]. Alkyl-anthocyanin adducts are acetaldehyde bridged dimers of anthocyanins and condensed tannins and are of peculiar relevance since they show a violet color that strongly contributes to the red hue of red wines [116]. These pigments are indeed one of the fates of monomeric tannins in red wine [117]. The above-mentioned pigments may show different polymerization degrees at random [118]. Yet, in this sense, pyranoanthocyanin dimers are special pigments formed by binding of two pyranoanthocyanins by an ethyl bridge and have a particular turquoise color [97]. For the time being, these specific dimer have only been identified in aged Port wines [89].

The overall transformation of complex pigments derived from wine anthocyanins yield red wines with a generally brighter color by the presence of vitisins and additional darker notes from porstisins, pinotins, and other adducts. On regard of white wines, color change is not caused by anthocyanin pigments, but rather the formation of xanthylium derived pigments and salts [48]. As mentioned, these can contribute to a yellow coloring of the white wine and their accumulation is also related with higher browning of the wine [88]. Hence, the color of white wines is dependent on the formation of these xanthylium salts and condensed tannins and flavonoids like (+)-catechin or (−)-epicatechin [84,119]. In this case, degradation of flavonoids has been related to a higher browning index because the formation of brown pigments is increased.

### 4.2. Aldehydes

Acetaldehyde, as the main aldehyde formed as a direct result of the oxidative chain reaction, is also the major aromatic aldehyde present. For this, is used as a marker of oxidation in line with degradation of SO_2_. Its aromatic behavior however, is to provide fruity aroma at low concentrations (≈30 mg/L) and rotten-like flavor at higher levels (≈100 mg/L) [120]. Moreover, as stated before, it is heavily involved in several parallel reactions taking place during bottle aging. For this, it has a pivotal role, while nonetheless being a marker to be controlled to avoid wine oxidative spoilage. In particular, it reacts very rapidly with SO^−3^ to produce an insoluble, less aromatic disulfite adduct [100]. For this reason, if SO_2_ is not added to the wine, acetaldehyde will probably be the predominant aroma [30]. Several other aldehydes, besides being related to wine oxidation, may provide mixed contributions to aroma, depending if their levels are below or above the perception threshold.

Thus, many aldehydes like octanal, nonanal or decanal, which are known as desired aromatic compounds, will present unpleasant odors because in oxidized conditions their levels may highly exceeds perception threshold [73]. Other aldehydes like phenylacetaldehyde, on the other hand, provide a honey-like aroma, which is relevant towards improving the aromatic profile of wines [121]. Aldehydes transferred from the barrel during oxidative aging, like furfural, show a decreasing trend during oxidative aging as they are degraded or react with other compounds like quinones. Regarding furfural, a key desired aromatic aldehyde, it tends to degrade during bottle storage as it reacts with other wine components, contributing the formation of xanthylium cations or producing aromatic thiols [84,108].

### 4.3. Other Compounds

Other relevant compounds affected by bottle storage and that contribute to the final profile of the wine will be summarized. These are tannins, nosiroprenoids, terpenols, and some thiols.

As mentioned, tannins in wine are mainly condensed tannins (also called procyanidins) derived from the grape, which are originated in the seeds and skins [122]. The main forms are polymers of flavan-3-ols [(+)-catechin, (−)-epicatechin, (−)-epigallocatechin, and (−)-epicatechin-3-*O*-gallate] with C_4_-C_6_ or C_4_-C_8_ linkages and monomeric units [123]. Tannins contribute to the dark color and astringency sensation of wine and through bottle storage may be subjected to hydrolization, freeing their flavanol subunit and ethyldiene bridged flavanol-phloroglucinol, subsequently hydrolyzed to ethyldienediphloroglucinol as residue [124]. Foremost, this liberation of flavanols leads to their availability for further reaction with aldehydes and anthocyanins to form anthocyanin/pyranoanthocyanin pigments, as well as a progressive loss of said astringency and dark color of the wine [125]. Nevertheless, tannins can also be repolymerized by H_2_O_2_ because of oxidation and, if they increase excessively in size, will sediment in the bottom of the bottle. In fact, if the wine contains great amounts of tannins that may not be sufficiently condensed by oxidation, they can be removed by enzymatic or gelatin fining processes that ease their precipitation [79]. Although both pathways may take place simultaneously ate the beginning of storage, with longer storage periods, tannins tend to degrade and significantly lower their repolymerization, which leads to reduced levels over time [126].

Norisoprenoids are also greatly important towards profiling wine’s aroma [14]. The norisoprenoid 1,1,6-trimethyl-l,2-dihydronaphthalene (TDN), shows a cooked meat/kerosene flavor and is generally unpleasant, being a marker of premature oxidation of wines [106]. However, at low concentrations it has been accounted for a caramel aroma and is a major aromatic compound in Riesling wines that has been determined to form by acid hydrolysis [127]. Although fairly recognized in this type of wines, TDN appears because of bottle storage regardless of the grape variety. Conversely, the norisoprenoids β-damascenone and β-ionone also appear to increase in bottle aging. These norisoprenoids are the result of degradation of grape carotenes and mainly appear during alcoholic fermentation, but with bottle storage, further oxidative degradation of these pigments lead to the liberation of these compounds [105]. Both norisoprenoids have a floral, fruity flavor and interestingly, β-damascenone has been reported to increase the pleasant aromatic sensation of other compounds present in wine [104].

Terpene alcohols (terpenols) derived from monoterpenes are very have an important role bottle aging aroma, as well as a shifting behavior in their concentration. The main terpenols in wine are geraniol, linalool, and α-terpineol [128] Albeit most aromatic terpenols originate from terpenoids in the grape skin, they can be newly formed during aging in bottle by acid-catalyzed hydrolysys from geraniol to linalool and/or α-terpenol through carbocation intermediates [102]. In the same way, it has been found that linalool may give place to geraniol by reduction with an hydroxyl anion [101] Linalool and α-terpineol have been reported to steadily increase at 18 months of storage but then show significant degradation after 24 months in Treixadura wines [129]. This kinetic behavior of terpenols has been also reported in many types of wines [130]. A possible explanation as to why their levels decrease in prolonged storage could be depletion of antioxidants and subsequent change of pH, making difficult these acid hydrolyzations.

Regarding thiols, the great majority of aromatic thiols are formed in alcoholic fermentation, being varietal thiols the most relevant [131]. Thus, the main task of bottle storage towards these thiols is to preserve them from degradation. Yet, during bottle aging some thiols that contribute to aroma may form. Some of these have deemed to be benzenemethanethiol and 2-furanmethanethiol, which have a perception threshold as low as < 1 ng/L. Benzenemethanethiol is characterized to have a “flint” and “roast” aroma [132]. Based on observations, it has been proposed by Tominaga et al. that benzaldehyde is a precursor for this thiols indicate that it reacts with free sulfur in the bottle as a decrease in benzaldehyde was correlated with higher levels of benzenemethanthiol [110]. 2-furanemethanethiol is a very odoriferous volatile thiol that gives off a roasted coffee aroma. The mechanisms responsible for 2-furanmethanethiol formation in wine have not yet been completely elucidated, but its production from yeast metabolism has been well described as well as a accounting for higher concentrations with the diminishing of furfural [109]. Nonetheless, Tominaga et al. observed that its content in champagnes increases in proportion to bottle aging time, along with a decrease in furfural [108]. Other thiol worth mentioning is 2-methyl-3furanthiol, with a “cooked meat” odor, and is generally found in smoked meats and also in wine in which provides a “toasty” note [133]. A general overview of the shifting in concentration of relevant wine compounds is summarized in Table 3.

## 5. Undesired Chemical Changes

### 5.1. Excessive Oxidation

Excessive oxidation, whether as result of high OTR during storage or uncontrolled storage conditions that may induce oxidation, can negatively affect the wine. Main symptoms of excessive oxidation are changes of color, appearance of compounds with “oxidative” aromas, and loss of varietal aromas [24]. Color changes are characterized as “browning” of the wine, a particularly noted fault in white wines that leads them to lose transparency or even obtain an unappealing color. Noted exceptions in white wines are white ports and sherry wines [48]. A main contributor to wine browning are increased concentrations of xanthylium cations that not only provide themselves a yellow coloration, but with further oxidation can be degraded to polymeric pigments exhibiting brown color [88]. Yet, the structure of said brown pigments formed in non-enzymatic reactions is still unknown as well as their specific source and synthesis pathways [118]. Nevertheless, their appearance has been related to diminishing levels of some possible precursor molecules such as flavonoids or anthocyanins as well as increasing levels of xanthylium salts [145].

Regarding the impact on aroma, excessively oxidized wines tend to exhibit an oxidation of ethanol resulting in overproduction of acetaldehyde and acetic acid, masking the wine aroma and giving an “oxidation” odor [37]. Acetic acid may be produced during alcoholic fermentation as a result of acetic acid bacteria metabolism but also by further oxidation of acetaldehyde in the bottle [100]. Acetaldehyde, besides being involved in many chemical reactions, (i.e., formation of puranoanthocyanins), is constantly formed by oxidation of ethanol and strongly contributes to oxidative change of wine and has the potential of becoming a dominant aroma over time. Moreover, several undesired aldehydes are formed as a result of oxidative imbalance and Strecker degradation of amino acids by quinones [70]. These are mainly identified as methional, 2-methylbutanal, phenylacetaldehyde, isobutyraldehyde, and isovaleraldehyde [100]. Methional is the foremost aldehyde identified in oxidized wines that gives off an aroma described as “boiled potatoes” [146]. The second major aromatic aldehyde is phenylacetaldehyde, formed by Strecker degradation of phenylalanine [70]. Differently from methional, as mentioned, phenylacetaldehyde may present sweet, floral fragrance at low concentrations, but “mossy” or “green” aroma at higher levels, which are prone to appear in highly oxidized wines [121]. On top of that, many esters synthesized during bottle aging are involved in the appearance of undesired aromas. Such is the case of acetates, formed by reaction of aldehydes with other molecules. The main ester in wine is ethyl acetate, synthesized from acetaldehyde and ethanol that haves a highly unpleasant “nail polisher” aroma [136].

Worth mentioning is the furan sotolon. Sotolon is a very powerful odorant that smells of “curry”, “roasting”, and caramel, whereas at higher levels can be responsible of a “rancid” odor [105]. It is mainly formed during wine maturation under a yeast film, but during bottle aging it can also be produced by condensation of α-ketobutyric acid and acetaldehyde and alternatively, degradation of ascorbic acid by ethanol [107]. As its levels during bottle aging are highly relying on the oxidative status of the wine, and at highly oxidation degrees causes a strong off-odor, it is considered as a marker of premature oxidation alongside TDN [147]. Other chemical group affected by excessive oxygen are volatile thiols are recognized for being important compounds affecting wine’s aroma in a strong manner. As mentioned earlier, varietal thiols are carried from alcoholic fermentation and barrel aging to the bottle, being very sensible to oxidation [148]. A relevant aspect of these compounds is that they exhibit perceivable odors at trace amounts of µg/L [131]. Main varietal thiols are the mercaptans 4-methyl-4-sulfanylpentan-2-one, 3-sulfanylhexyl acetate, 4-methyl-4-sulfanylpentan-2-ol, 3-sulfanylhexan-1-ol, and 3-methyl-3-sulfanylbutan-1-ol. 3-sulfanylhexan-1-ol is considered the most relevant, providing an appreciated sweet grape-like flavor [109]. Being first described in Sauvignon Blanc, these compounds are of great importance in conferring pleasant “box”, “fruity”, and “floral” aroma to wines [149]. Their oxidative degradation as a result of prolonged storage leads to a marked loss of aroma that coupled with the appearance of other undesired compounds may significantly harm the quality of the wine [37]. For this reason, their levels are measured to determine not only the loss of desired aromas in highly oxidized wines.

Eventually, differences in composition and level of these compounds are related to the oxidation degree, grape variety, and desired type of wine. For example, fortified Sherry and Port wines are highly oxidized in comparison to other wines and account for high levels of acetaldehyde or sotolon, having a desired aroma in this case [105].

### 5.2. Reductive Faults

Reductive faults comprise sulfur-based compounds like hydrogen sulfide (H_2_S), mercaptans and disulfides [59]. The most important and common faulty mercaptan is methanethiol (MeSH) [150]. Their presence at very low concentrations (1–3 µg/L) confer the wine highly unpleasant off-odors like “rotten egg” or “rotten cabbage” and mask desired aromas [61]. These compounds are present in bound and free forms, and being volatile in nature, their free odorous form is in the vapor phase [151]. Some H_2_S can be present in the wine prior to bottling by reduction of sulfated pesticides used in the vineyard or as a result of barrel aging storage, but is mainly produced by yeasts metabolism during fermentation [59]. Moreover, another source of H_2_S is SO_2_, as the degradation of H_2_SO_4_ frees sulfur that can be subsequently reduced to H_2_S while the oxygen forms H_2_O_2_ [62].

Their levels are reported to be related to an insufficient oxidation of wines and appear at higher levels in anoxic bottle aging, i.e., in bottles enclosed with screw caps. Wines with higher copper concentrations at the end of bottle storage have been found to contain greater levels of H_2_S and MeSH in several experiments [61]. A traditional chemical solution to reduce the appearance of these molecules has been copper fining, which implies the addition of low quantities of copper (≈1 mg/L) prior to bottling [62,152]. This paradoxical effect of copper has been correlated in function of its concentration and oxygenation during storage, being suggested that in high concentrations and low oxygenation, free copper ions are released from their bound form with sulfur species [153]. Despite the mentioned evidence that copper induces further oxidation by iron and at high concentrations, it is also related to higher final levels of reductive characters, copper fining remains a widely extended technique [152]. Its usage by winemakers may yield mixed results; for it has been observed that although copper fining lowers appearance of these compounds for short periods of time, may result in a greater production of them after long storage periods [154].

**Figure 4:**
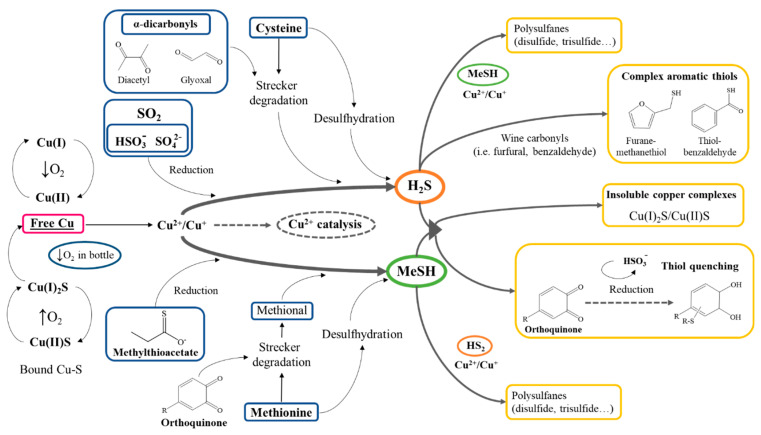
Formation and fate of SH_2_ and MeSH involving copper catalysis. Unknown precursors are not shown in the diagram. Adapted [73,157].

In a recent experiment conducted by Franco-Luesma and Ferreira, release of H_2_S and MeSH from bound sulfur/copper complexes was suggested as a major pathway that leads to the accumulation of these free reductive characters in red wines during anoxic aging [155]. In another experiment by the same team [156], the use of micro-oxygenation during fermentation reduced the levels of free forms of H_2_S and MeSH, but not of the complexes, that dissociate into free forms in low oxygen conditions. These facts would indicate that oxygenation ameliorates the formation of free H_2_S and MeSH, but will be formed nonetheless during bottle storage and with more intensity in anoxic conditions [152]. The “de novo” production and accumulation of reductive compounds is hypothesized to be the result of various possible reactions, like reduction of sulfate or sulfite, hydrolysis of thioacetates or Strecker degradation of the sulfur-rich amino acids methionine and cysteine [157]. The Strecker degradation reaction with cysteine is carried out by α-dicarbonyls present in the wine like glyoxal and diacetyl [158]. Additionally, cysteine and methionine can suffer a desulfuhydration process catalyzed by copper that leads to the liberation of their sulfur group [154]. On this matter, ortho-quinones act as indirect inducers of their formation as they promote the Strecker degradation of amino-acids and free undesired aldehydes (i.e., methional from methionine) in the process [70]. Yet, given the nucleophilic nature of ortho-quinones, they can also capture thiol compounds. The binding between orthoquinones and thiols has been studied to be more reactive towards H_2_S than desirable aromatic thiols, meaning that they may lower levels of these compounds before causing a loss of varietal thiols [140]. It has also been researched that H_2_S, MeSH and/or other thiols like glutathione can bind together with Cu^2+^, reducing the metal ion and yielding polysulfanes that further contribute to the “reductive” aromatic fault [152]. The discerned complex interactions of copper and these undesired thiols is summarized in Figure 4.

Although how and when many of these reactions take place remains to be explained, evidence suggests that the best way to avoid appearance of free volatile sulfated compounds would be the constant ingress of oxygen [154]. For this reason, the stopper and its properties play yet another critical role.

## 6. Main Factors Affecting Quality during Storage

Proper storage of wine bottles is a key element of preserving the wine, because as with any other food and beverage, temperature, humidity, and light exposure are known promoters of oxidation. Yet, these environmental conditions also exert influence on the stopper gas permeability and mechanic properties [24]. Therefore, control over storage conditions bears an increased hold on the bottle aging process. Besides, bottled wines may develop faults non-reliant on oxidation or microbiological contamination, but compounds transferred from cork stoppers or the wine cellar environment [159].

### 6.1. Temperature

Besides uncontrolled oxygen ingress, fluctuating temperature is the main risk associated with wine spoilage. A temperature interval of 15–17 °C is acknowledged as optimum for wine preservation. Lower temperatures will slow the aging process, whereas intuitively, elevated temperatures (>20 °C) will accelerate oxidative reactions [147]. However, because it is a critical parameter to withhold quality of the wine and affects both the stopper and wine components, shifting temperatures are considered a great issue. These shifting temperatures will likely take place during retailer storage and transportation. Major undesired features from elevated storage temperature include degradation of anthocyanin pigments, formation of xanthylium salts, degradation of aromatic compounds (thiols, esters, aldehydes) and deposition of protein haze, caused by denaturation of wine proteins [160]. This is translated in “browning” of color and a cloudy appearance caused by haze. Both browning and haze are easily perceivable in white wines, given their absence of anthocyanins and thus color that could mask these unappealing visual changes [161]. The loss of fruity desired aromas is also weighted by the appearance of undesired oxidative aroma compounds such as TDN or acetaldehyde, reflecting the loss of quality [162].

Using screw caps appears to ameliorate the accelerated oxidation induced by elevated temperature, as it limits the available oxygen [44]. This property of screw caps has been explored as a mean to quicken the long storage times needed for bottle aging of red wines as “accelerated aging”. As wine bottles are hermetically sealed, temperature is the sole inducer of oxidation, but this option is [106]. Overall, a slightly low, constant temperature is considered best to storage wines both for their aging and preservation, which justifies their storage in wine cellars.

### 6.2. Light Exposure

Light and especially UV-light is well-known as an inducer of oxidation and production of reactive oxidative species. As in the case of many other beverages and foods, light promotes oxidation in wine, fastening the oxidation rate [147]. In the case of wine, iron light-induced ionization has been proposed to act as the main promoter of oxidation by this source in relation to the pivotal role that plays in its chemical oxidation. Especially iron-tartrate conjugates for their known role in oxidation, have shown to significantly increase browning [163]. This may result in the typical features of excessively oxidized wines, such as a loss of aromatic compounds, browning and appearance of oxidative aromas, (acetaldehyde, acetic acid, and sotolon) which has been observed in wines stored under light exposure [74]. Using darkened glass bottles as containers to preserve wine is justified, since it reduces the incidence of light, yielding lower degradation values of aromas and lesser browning [164]. Yet not only UV-light induces browning, but also artificial light. Ferreira lima et al. tested the preservation of Goethe wines under “supermarket” conditions (25 °C/2500 lumens/12 h) for 10 months, finding a highly-increasing browning and faster degradation of phenolic acids and flavonoids [165]. In a very recent study, the influence of antioxidants towards light-induced oxidation further confirmed that ascorbic acid and/or SO_2_ delay light-induced oxidation [141]. Given the known evidence, minimizing exposure to light is held as a vital condition to properly store wine.

### 6.3. Humidity

Humidity tends to lower the permeability to oxygen of cork stoppers. As before mentioned, drier stoppers tend to shrink and show a generally higher permeability to oxygen. As such, a ≈70% relative humidity is accepted as optimal to storage wine bottles [24]. Hence, to mindfully control and/or extend permeability properties of porous stoppers, constant high humidity conditions are maintained in winecellars. This explains why most ancient winecellars are built underground, since it conforms the optimum environment in term of light exposure, temperature and humidity. Even so, high humidity levels can promote the growth of spoilage molds in winery cellars [166]. For this matter, not only management of humidity is necessary but also a thorough sanitation of the cellar environment to avoid undesired development of spoilage microorganisms.

### 6.4. Position

Wine bottles have traditionally been stored in horizontal position for space needs but also to limit excessive oxidation. From a theoretical point of view, the gas transference rate will be faster when the stopper is in contact with the gas phase in the bottle headspace and slower if in direct contact with the wine. However, experimental research has yielded unclear results. Mas et al. found that horizontal position resulted in lower oxidative parameters (i.e., acetaldehyde, acetic acid, anthocyanin degradation) comparing to vertical storage [167]. Hernanz et al. results showed a slight increase in oxidized PC in vertically stored white wines [168]. On the other hand, Lopes et al. and Skouroumounis et al. did not find significant alterations by position after 2 and 5 years of storage, respectively [46,83]. Collected data would suggest that horizontal position during storage may, at least, slightly reduce oxidation in wine while also being convenient for storage space management. Yet, as it seems that the contribution of position is reliant on the stopper properties and wine composition, more research on this topic is required.

### 6.5. Environmental Off-Flavors

The most relevant exogenous compounds liable of off-odors and flavors are caused by haloanisoles and bromohanisoles of which the major representatives are 2,4,6-trichloroanisole (TCA), 2,4,6-tribromoanisole (TBA), respectively. Geosmin, guaicol or 1-octen-3-one are also responsible for exogenous off-flavors. These compounds can produce sensory spoilage of the wine in concentrations as low as 2 ng/L, causing a moldy, earthy or “cardboard” taste. TCA and TBA are produced by naturally present microorganisms in cork oak bark and can be transferred to the bottle if the stopper has not been properly treated or sanitized. Hence, their off flavor caused by their presence is called “cork taint”. By other hand, geosmin, or guaicol are the result of bacterial or fungal contamination of the wine and/or the stopper. To avoid the spoilage caused by these compounds, preventive measures to avoid contamination must be taken during the winemaking process, as well as a proper storage of the bottles in a likewise sanitized environment [169,170]. Nevertheless, sanitizers used must be free of chlorine, since this chemical might bind with cork components to form TCA and TBA [171]. An alternative way to partially avoid the appearance of the responsible microorganisms is using synthetic stoppers or screw caps, since are easily sterilized and do not promote the growth of the microorganism responsible for these compounds. Yet, it must be considered that synthetic stoppers may allow leak of these compounds from the environment to the wine, given their high porosity [56]. An additional and extended alternative is the encapsulation of the closure with a plastic or metallic foil, which has proven efficient to avoid contamination of the stopper by these microorganisms and most importantly, from TCA and TBA ingress [53].

## 7. Conclusions

Summarizing collected data, bottle aging and storage of wine is an important albeit complex and sensible process that greatly influences the features of the final product. During storage, deep changes in aroma, color stability, appearance, and mouthfeel take place and define the quality of wine, which, in turn, impacts consumer preference and appreciation. The wide variety of available wines involves different aging times and conditions for each type of wines, also relying on the vinification techniques they have been subjected to. Foremost, the fitness of a wine to be aged is highly related to its composition of phenolic compounds and overall oxidative stability. On this matter, proper storage conditions and careful selection of the stopper used to enclose the bottle is a key issue that greatly determines adequate aging and therefore, a desired outcome for winemakers. A mindful control of a wine’s oxidative balance and oxidation will also allow to avoid the arise of undesired compounds responsible for wine spoilage, whether because of excessive or insufficient oxygenation. As knowledge on the complex chemistry of wine through aging has increased in recent years, further research could shed light in still unknown pathways and relevant mechanisms involved.

## Figures and Tables

**Table 1:** Mean oxygen transfer rate of several types of stoppers *.

Type	Size Range (Length × Diameter) (mm)	Oxygen Transfer Rate (mg/Year) **	References
Natural cork	49 × 24	5.5	[28]
	45 × 24	6.37	[45]
	44.7 × 24	2.03	[46]
	45 × 24	14.25	[34]
	45 × 24	3.65	[37]
Agglomerated cork	45 × 24	2.62	[45]
	45 × 24	1.8	[47]
	Not mentioned	0.78	[48]
	45 × 24	1.68	[49]
	45 × 24	2.68	[49]
Technical stopper	43.6 × 23.7	2.03	[46]
	44 × 24	1.8	[29]
	44 × 24	1.9	[47]
	49 × 24.2	0.61	[43]
	49 × 24.2	0.38	[43]
Molded Synthetic	44.6 × 21	6.8	[46]
	44.7 × 21	20.8	[45]
	43 × 22	6.5	[47]
	Not mentioned	22.25	[42]
	Not mentioned	6.95	[42]
Extruded synthetic	43 × 22	13.65	[45]
	23 × 38	3.28	[50]
	23 × 38	6.57	[50]
	38 × 24	9.38	[37]
	38 (length)	4.34	[25]
Screw cap	31.5 (diameter)	2.52	[29]
	31.5 (diameter)	1.82	[29]
	Not mentioned	0.23	[46]
	60 × 30	0.5	[47]
	Not mentioned	0.31	[37]

* Data shown indicate measures taken on the closure alone with appropriate seals and equipment (i.e., metal tubes/rings), chemical oxygen determination methods in wine or data provided by producers. ** Oxygen transfer rate units from literature have been calculated and/or extrapolated to mg year^−1^ for practical purposes, when necessary.

**Table 2:** Major compounds of interest produced during bottle aging.

Type	Name	Pathway(s)	Features	References
**Pigments**	Vitisin A	Condensation with pyruvic acid	Bright red-orange color, stable	[89]
	Vitisin B	Condensation with acetaldehyde	Bright red-orange color, stable	[90]
	Pinotin A-like pyranoanthocyanins	Binding with hydroxycinnamic acids and aldehydes	Red-orange color, stable	[91]
	Portisin A, portisin B, portisin C	Binding with flavanols through vinylphenols	Bluish color, stable	[92,93]
	Anthocyanin dimers and trimers	Polymerization	Dark-red color, stable	[94]
	Anthocyanin-flavanyl adducts	Binding with vinylflavanols	Bright red-orange color, stable	[95]
	Anthocyanin-alkyl-flavanol adducts	Binding with an alkylflavanol through aldehydes	Purple color	[96]
	Pyranoanthocyanin polymers	Binding of vitisin A with a methyl-pyranoanthocyanin	Light-blue/turquoise color, stable	[97]
	Oxovitisins	Hydrolization of Vitisin A	Bright yellow color, stable	[98]
**Aldehydes**	Acetaldehyde	Ethanol oxidation	Precursor of polymerized pigments and tannins, fruity flavor at low levels, main oxidation marker	[99]
	Phenylacetaldehyde	Strecker degradation of phenylalanine	Sweet, honey-like aroma at low ct.; mossy aroma at high ct.	[100]
**Terpenols**	Geraniol	Hydrolysis from linalool	Floral aroma	[101]
	Linalool	Hydrolysis from geraniol	Rose aroma	[102]
	α-terpineol	Hydrolysis from geraniol/linalool	Floral aroma	[103]
**Norisoprenoids**	β-damascenone	Oxidative cleavage of neoxanthin/allene terpene	Rose aroma, can enhance perception of other fruity aromas	[104]
	β-ionone	Oxidative cleavage of β-carotene	Cooked apple aroma	[105]
	TDN	Decarboxylation with acetaldehyde	Kerosene-like aroma	[106]
**Furans**	Sotolon	Degradation of ascorbic acid/α-ketobutyric acid	Curry, spicy flavor	[107]
**Thiols**	2-furanmethanethiol	Proposed formation from furfural	Toasted coffee aroma	[108,109]
	Benzenemethanethiol	Sulphuration of benzaldehyde	Flint, roast aroma	[110]

**Table 3:** General tendency of increasing and decreasing relevant compounds during bottle aging.

Compounds.		Increase	Decrease	Ref.
***Pigments***	Monomeric anthocyanins		*	[134]
	Polymeric anthocyanins	*		[114]
	Pyranoanthocyanins	*		[134,135]
	Anthocyanin-flavanol polymers	*		[135]
	Anthocyanin-alkyl adducts	*		[134]
***Volatile phenolics***	Guaiacol		*	[136]
	Syringol	*		[137]
	Eugenol	*		[138]
***Flavonoids***	(+)-catechin		*	[136,139]
	(−)-epicathechin		*	[66,136]
	Kaempferol		*	[66,136]
***Thiols***	Varietal thiols (3SH, 4MSP, etc.)		*	[140]
	Complex thiols (2FMT, BMT, etc.)	*		[108]
***Phenolic acids***	Caffeic acid	*		[141,142]
	Gallic acid	*		[141,142]
	p-coumaric acid	*		[143]
	Hydroxycinnamic acids		*	[66]
	Syringic acid	*		[136]
	Caftaric acid		*	[139,142]
	Coutaric acid		*	[143]
	Fertaric acid		*	[142]
	Ferulic acid	*		[139]
***Esters***	Ethyl acetate	*		[136]
	Acetoine	*		[129]
***Terpenols*** **^1^**	Linalool	*		[144]
	Geraniol	*		[101]
	α-terpineol	*		[130]
***Aldehydes***	Acetaldehyde	*		[136]
	Methional	*		[70]
	Phenylacetaldehyde	*		[121]
	Octanal	*		[42]
	2-nonanal	*		[42]
	Decanal	*		[31]
	Furfural		*	[121]
***Norisoprenoids***	β-damascenone	*		[104]
	β-ionone	*		[37]
	TDN	*		[106]
***Tannins***	Monomeric flavan-3-ols		*	[126]
	Epigallocatehcin		*	[126,144]
	Gallocatechin		*	[144]
	Vescalagin		*	[125]

^1^ Tendency of increase during first year of aging, followed by decrease. 3SH: 3-sulfanylhexanol, 4MSP: 4-methyl-4-sulfanylpentan-2-one, 2FMT: 2-furanmethanethiol, BMT: Benzenemethanethiol.

## Abbreviations

PC	Phenolic compounds
OTR	Oxygen transfer rate
SO_2_	Sulfide dioxide
HSO_3_^−^	Bisulfite anion
SO_4_^2−^	Sulfate
H_2_SO_4_	Sulfuric acid
H_2_S	Hydrogen sulfide
MeHS	Methanethiol
H_2_O_2_	Hydrogen peroxide
HO^−^	Hydroxyl radical
TDN	1,1,6-trimethyl-l,2-dihydronaphthalene
TCA	2,4,6-trichloroanisole
TBA	2,4,6-tribromoanisole
